# Ammonium sulfate denatures transport medium less dependent on guanidinium isothiocyanate and enables SARS-CoV-2 RNA and antigen detection compatibility

**DOI:** 10.3389/fmicb.2024.1384991

**Published:** 2024-05-03

**Authors:** Ge Liu, Jiapeng Xu, Yuanyuan Huang, Wei Ye, Jieyu Li, Ran Yan, Qiting Luo, Xinrui Zhou, Yingna Cai, Hanfang Jiang, Xiujing Lu, Kai Zheng, Zhendan He, Qinchang Zhu

**Affiliations:** ^1^College of Pharmacy, Shenzhen Technology University, Shenzhen, China; ^2^School of Pharmaceutical Sciences, Shenzhen University, Shenzhen, China; ^3^Clinical Laboratory, Shenzhen Children's Hospital, Shenzhen, China; ^4^GBCBIO Technologies Inc., Guangzhou, China

**Keywords:** COVID-19, RNA detection, antigen detection, denaturing transport media, guanidinium isothiocyanate, ammonium sulfate

## Abstract

**Introduction:**

Rapid identification of infected individuals through viral RNA or antigen detection followed by effective personal isolation is usually the most effective way to prevent the spread of a newly emerging virus. Large-scale detection involves mass specimen collection and transportation. For biosafety reasons, denaturing viral transport medium has been extensively used during the SARS-CoV-2 pandemic. However, the high concentrations of guanidinium isothiocyanate (GITC) in such media have raised issues around sufficient GITC supply and laboratory safety. Moreover, there is a lack of denaturing transport media compatible with SARS-CoV-2 RNA and antigen detection.

**Methods:**

Here, we tested whether supplementing media containing low concentrations of GITC with ammonium sulfate (AS) would affect the throat-swab detection of SARS-CoV-2 or a viral inactivation assay targeting coronavirus and other enveloped and non-enveloped viruses. The effect of adding AS to the media on RNA stability and its compatibility with SARS-CoV-2 antigen detection were also tested.

**Results and discussion:**

We found that adding AS to the denaturing transport media reduced the need for high levels of GITC, improved SARS-COV-2 RNA detection without compromising virus inactivation, and enabled the denaturing transport media compatible with SARS-CoV-2 antigen detection.

## 1 Introduction

The ongoing COVID-19 pandemic has resulted in more than 772 million infections and nearly 7 million deaths worldwide since its emergence in late 2019 (Wang et al., 2020; WHO, 2023). The WHO Director-General declared an end to the COVID-19 public health emergency of international concern (PHEIC) on May 5, 2023, but this does not mean the pandemic is over. Over 850,000 new COVID-19 cases have been reported worldwide in the past 28 days, a 52 percent increase compared to the previous period (WHO, 2023). COVID-19 is still a global threat, and new variants of the coronavirus could still emerge. Although several vaccines (e.g., mRNA vaccines, inactivated vaccines, and viral vector vaccines) have been conditionally approved in many countries (Sharma et al., 2020; Li et al., 2021) and several antiviral drugs have been approved in clinics (Españo et al., 2021; Wen et al., 2022), there is currently still no effective way to stop the spread of COVID-19. Before all susceptible populations are fully protected, dealing with a pandemic by controlling the infection sources and blocking the transmission routes, which rely on rapidly identifying SARS-CoV-2 infected individuals and isolating them from the population remains extremely important. Nucleic acid-based methods are highly sensitive for detecting SARS-CoV-2, especially in early infection (Tsang et al., 2021). Such methods have played irreplaceable roles in the mass screening for SARS-CoV-2 infection in the early epidemic stage. Large-scale nucleic acid testing has been implemented following local case reports where possible community transmission is indicated, and this approach has supported China’s sustained containment of COVID-19 (Li et al., 2021). Wuhan (Hubei Province) performed city-wide mass screening using reverse-transcriptase quantitative PCR (RT-qPCR) testing for nearly 10 million people over 10 days (Cao et al., 2020), and Qingdao (Shandong Province) tested 10.9 million people for SARS-CoV-2 RNA in 5 days (Xing et al., 2020). Besides nucleic acid detection, rapid antigen detection (RAD) is also recommended by WHO as a screening method for COVID-19. RAD mainly targets the N-protein antigen locus of SARS-CoV-2, which is relatively quick, inexpensive, and laboratory-independent, but the overall sensitivity of RAD is lower than RT-qPCR (Pavia and Plummer, 2021; Weitzel et al., 2021). Thus, RAD has been suggested as an adjunct to nucleic acid detection for COVID-19 diagnosis (Li et al., 2023).

Large-scale nucleic acid testing involves large-scale specimen collection and transportation. Nasopharyngeal and throat swabs are usually used in the specimen collection for SARS-CoV-2 RT-PCR testing (Tsang et al., 2021). After collection, specimens are generally preserved and transported in non-denaturing or denaturing media. Non-denaturing media, such as conventional viral transport medium (VTM) and Amies transport medium, mainly contain saline buffer and antibiotics (Johnson, 1990; Smith et al., 2020; Penrod et al., 2021). Non-denaturing media are used to maintain the integrity and infectivity of the viruses in the specimen. Therefore, non-denaturing media are used to detect viral nucleic acids and RAD or for culture-based viral detection. However, laboratory personnel risk infection when handling infectious specimens during the testing process, especially during large-scale testing (Karthik et al., 2020). Denaturing transport media usually contain guanidinium isothiocyanate (GITC) or other virus-inactivating denaturants, which is why they are only suitable for nucleic acid detection. One advantage of denaturing transport medium is its potential to reduce the risk of infection of laboratory personnel and improve SARS-CoV-2 RNA detection (Welch et al., 2020; Carvalho et al., 2021; Erster et al., 2021). Both denaturing and non-denaturing viral transport media are widely used (van Bockel et al., 2020; Welch et al., 2020; Penrod et al., 2021), and the former are recommended in China for large-scale nucleic acid testing (National Health Commission of the People’s Republic of China, 2020). Although various commercially available and in-house denaturing viral transport media exist, no uniform formulation standards exist. The core component of denaturing viral transport media is usually GITC, whose concentrations are mostly between 30 and 50% (2.5–4.2 M) (Welch et al., 2020). GITC is a chaotropic agent with a strong protein denaturing function. It is commonly used as a nucleic acid protector during RNA and DNA extraction because it denatures RNases and DNases. GITC also deactivates many viruses, including SARS-CoV-2 (Pastorino et al., 2020; Welch et al., 2020; Westhaus et al., 2020).

However, with the growing demand for denaturing viral transport media in large-scale nucleic acid testing, a GITC supply shortage has occurred in China. Furthermore, the high GITC concentrations in the viral testing platforms can react with bleach (sodium hypochlorite) to produce harmful cyanide gas (Paik and Wu, 2005). On the other hand, although denaturing transport media that efficiently inactivate the virus could reduce the risk of infection during antigen assay performance, especially in point-of-care testing, there is a lack of denaturing transport media that is compatible with SARS-CoV-2 antigen detection (Zhou et al., 2021; Conzelmann et al., 2022). Therefore, optimizing the formulation by reducing the GITC content may be a way to help meet the demand for SARS-CoV-2 screening and reduce the testing costs and harm. Ammonium sulfate (AS) is a low-cost inorganic salt with high water solubility from its ionic nature. It is often used for protein precipitation and purification in the laboratory. High-concentration AS is also used to enhance RNA stability in tissue samples (Mutter et al., 2004; DAUM et al., 2011). Here, we report that reducing the amount of GITC in denaturing viral transport media while adding AS to them can improve the sensitivity of SARS-CoV-2 RNA detection without affecting the virus inactivation effect and enable the denaturing transport media compatible with SARS-CoV-2 antigen detection.

## 2 Materials and methods

### 2.1 Cells and viruses

Vero cells (ATCC, CCL-81) and RD cells (ATCC, CCL-136) were cultured in Dulbecco’s modified Eagle’s medium (Gibco, NY, United States) supplemented with 10% (v/v) fetal calf serum. SARS-CoV-2 pseudovirus (FNV-2019-nCoV-abEN, >10^8^ copies/mL) was purchased from Fubio Biological Technology Co., Ltd. (Hangzhou, China). Human coronavirus OC43 (HCoV-OC43) and herpes simplex virus type 1 (HSV-1, VR-733) originated from the American Type Culture Collection. Enterovirus 71 (EV71, C4 strain) was kindly provided by Dr. Tao Peng, Guangzhou Medical University, China. HSV-1 and EV71 were propagated and tittered in Vero cells, while HCoV-OC43 was propagated and tittered in RD cells. SARS-CoV-2 pseudovirus was used directly.

### 2.2 Denaturing solutions

Modified Primestore Molecular Transport Medium (MTM), which we used as a reference medium, contains 3 M GITC, 25 mM sodium citrate, 0.5% SLS (sodium lauryl sulfate), and 20 mM ethylenediaminetetraacetic acid. Based on the modified Primestore MTM, we prepared the denaturing transport medium with various GITC concentrations and/or AS as indicated.

### 2.3 SARS-CoV-2 RNA detection

A throat swab sample from a healthy person was collected in 4 mL saline, and the 100 μL of SARS-CoV-2 pseudovirus (>10^7^ copies) we added to the sample was mixed in by vortexing. Next, 200 μL of the SARS-CoV-2 pseudovirus-containing throat swab sample was added to 1 mL of each denaturing solution we prepared or to PBS. The solutions were kept at room temperature (RT) or 37°C, and RNA was extracted from them using the Virus DNA/RNA Kit (GBCBIO) after incubation for 24, 72, 120 h, or 5 days. RT-qPCR detection of SARS-CoV-2 RNA was performed in 20-μL reactions. The BeyoFast™ Probe One-Step qRT-PCR Kit (Beyotime) and primer and probe sequences targeting the ORF1ab gene and N gene were used following the protocols for COVID-19 Prevention and Control Guidelines (Seventh Version) issued by China’s National Health Commission. Each sample’s cycle threshold (Ct value) was recorded and used for cross-sample comparisons. Graphs and statistical analyses (multiple *t*-tests) were prepared with GraphPad Prism 8.0.2 (GraphPad Software, Inc., La Jolla, CA, United States). Statistical significance was determined using the Holm-Sidak method, with an alpha of 0.01.

### 2.4 Virus inactivation assay

The viral inactivation abilities of the denaturing solutions containing different GITC concentrations and/or AS were investigated using a plaque reduction assay. HCoV-OC43 (100 μL, 4.5 × 10^7^ plaque-forming units (PFUs)/mL), HSV-1 virus (4.135 × 10^8^ PFUs/mL) or EV71 virus (5 × 10^7^ PFUs/mL) was added to 0.9 mL of each denaturing solution, and each solution was mixed and incubated for 1 h at RT. After incubation, each mixture was serially diluted and filtered through Millex-GP 0.22 μm membrane filters (Millipore) and finally used in a plaque reduction assay using previously described procedures (Zhu et al., 2010, 2011).

### 2.5 RNA stability assay

1 × 10^7^ eukaryotic cells were harvested and subjected to total RNA extraction using the Total RNA Kit (GBCBIO). Next, 8 μL of the total RNA (about 1.0 μg/μL) was added to 50 μL of each denaturing solution, and each solution was mixed and incubated for 24 h at RT. After incubation, each mixture was analyzed by 1.2% (wt/vol) agarose gel electrophoresis in 1 × TAE buffer at 80 V for about 40 min and visualized by staining with 4S Green Plus Nucleic Acid Stain (BBI Life Sciences, China) and ultraviolet transillumination. In addition, the concentration of residual RNA in the mixture was determined by spectrophotometer based on the absorbance at 260 nm.

### 2.6 SARS-CoV-2 antigen detection

SARS-CoV-2 N recombinant protein (BBI Life Sciences, China) was diluted with PBS to 0.5 mg/mL, and then 2 μL of diluted N recombinant protein was added to 400 μL of each denaturing solution or the solution from 2019-nCoV Antigen Test Kit (colloidal gold method) (Wondfo, China). Each solution was mixed and incubated for 1 min at RT. After incubation, 100 μL of the mixture was dropped into the testing device from the antigen test kit. After waiting for 15 min, as recommended, read the results.

### 2.7 Statistics analysis

All the data presented herein are mean ± standard derivation values. Statistical comparisons were analyzed with a two-tailed Student’s *t*-test using GraphPad Prism version 8.0.2 for Windows (GraphPad Software, California, United States). ^**^indicates *p* < 0.01 and ^*^indicates *p* < 0.05. *p* values <0.05 were considered statistically significant.

## 3 Results and discussion

### 3.1 Effects of the test solutions on SARS-CoV-2 RNA detection

To evaluate the effect of adding AS to denaturing transport media on SARS-CoV-2 RNA detection, the experiments used a modified commercial denaturing transport medium containing 3 M GITC as the basic formula and reference reagent. To mimic the sampling of SARS-CoV-2, a throat swab sample from a healthy person was mixed with SARS-CoV-2 pseudovirus. It was then placed into denaturing media containing different concentrations of GITC and AS and incubated at room temperature or 37°C for 24, 72, 120 h, or 5 days as indicated. After RNA extraction, qRT-PCR on the SARS-CoV-2 ORF1ab and N genes was performed and comparatively analyzed.

**Figure 1:**
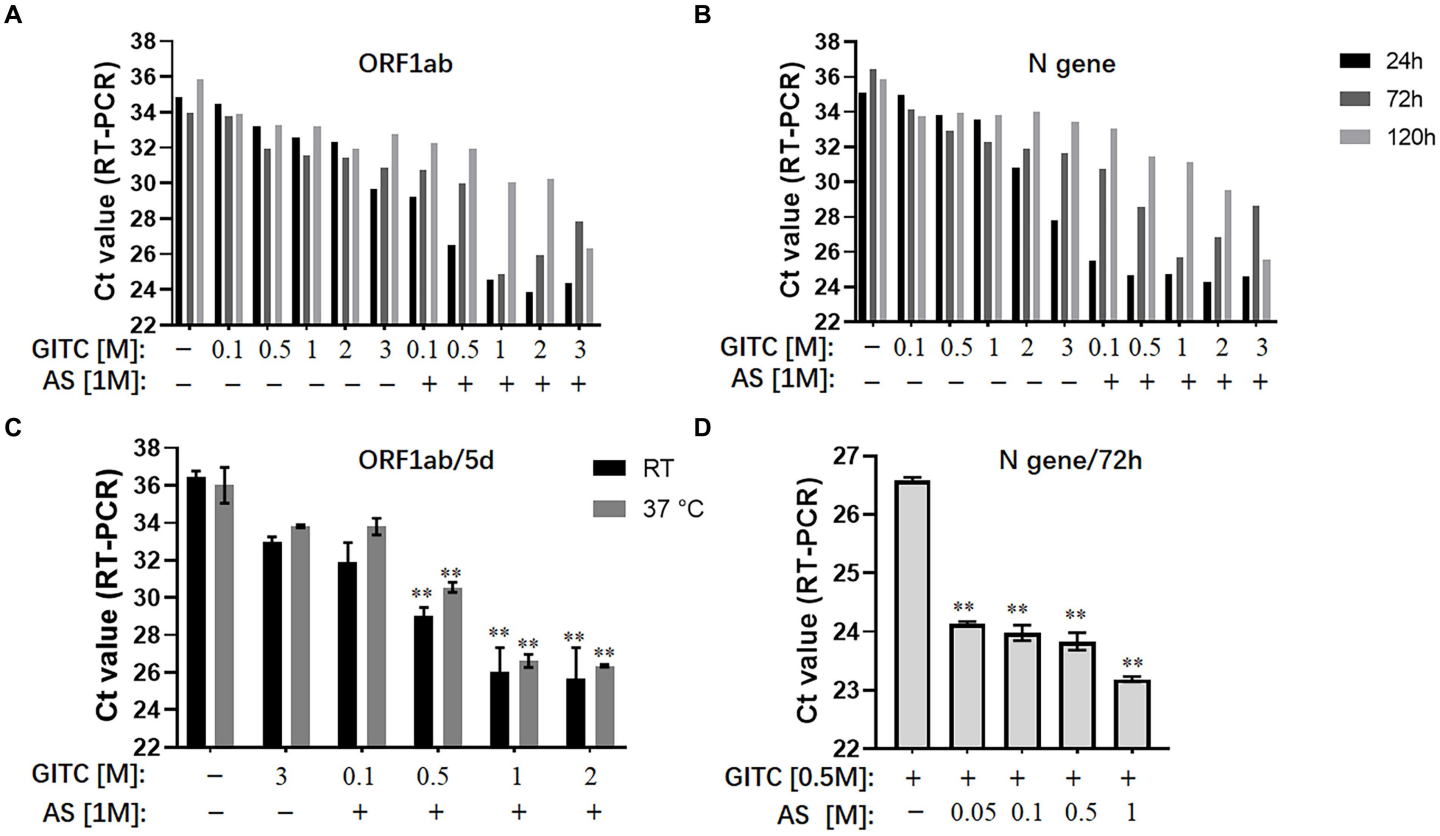
Effects of ammonium sulfate (AS) addition to denaturing transport media on SARS-CoV-2 RNA detection. **(A)** ORF1ab gene detection for various samples in denaturing media or PBS for 24, 72, and 120 h at room temperature (RT). **(B)** N gene detection for various samples in denaturing media or PBS for 24, 72, and 120 h at RT. **(C)** ORF1ab gene detection for various samples in denaturing media or PBS for 5 days at RT or 37°C. ^**^p < 0.01 vs. 3 M GITC group. **(D)** N gene detection for various samples in denaturing media across an AS concentration gradient for 72 h at RT. ^**^p < 0.01 vs. Control group. GITC, Guanidinium isothiocyanate. Samples without GITC and AS are the PBS controls.

The results showed that reducing the GITC concentrations increased the Ct values, but adding 1 M AS to the transport media reduced the Ct values of the ORF1ab gene and the N gene irrespective of the 24, 72, or 120-h incubation period (Figures 1A,B). A further assay on the samples incubated at RT or 37°C for 5 days revealed that adding 1 M AS to transport media containing lower GITC concentrations significantly reduced the Ct values when compared with PBS or the transport medium containing 3 M GITC (*p* < 0.01) (Figure 1C). A clear dose-dependent effect was observed when AS was added to each transport medium containing a fixed GITC concentration (Figure 1D). Because lower Ct values indicate higher qRT-PCR detection efficiency, these results suggest that adding AS to the transport media can reduce the amount of GITC required for sample denaturation and increase the detection efficiency for SARS-CoV-2 RNA.

Guanidinium isothiocyanate is commonly used at high concentrations in denaturing viral transport media (Pastorino et al., 2020; Welch et al., 2020). However, the sharp increase in demand for large-scale nucleic acid testing is causing a shortage of GITC during the ongoing COVID-19 pandemic. Another issue is that the high GITC concentrations in the viral testing platforms can react with bleach (sodium hypochlorite) to produce harmful cyanide gas (Zhu et al., 2011). Therefore, reducing the GITC concentrations in transport media is worthwhile. However, simply reducing its concentration in the transport medium decreased the detection efficiency of SARS-CoV-2 RNA (Figures 1A,B). Here, we showed that adding AS to the transport media not only recovered but also improved the detection efficiency when low concentrations of GITC were present. The use of AS to precipitate proteins out of solution is known, and AS has been reported to suppress more than 90% of RNase A activity (Allewell and Sama, 1974; Lader, 2001). Therefore, the improved RNA detection efficiency observed in this study possibly resulted from AS-induced denaturation of the RNase A protein, but further research is needed to confirm it.

In addition, adding AS to the viral transport medium did not affect the RNA extraction in this study. Guanidinium thiocyanate-phenol-chloroform extraction and solid-phase nucleic acid extraction are two commonly used methods for extracting RNA from samples. The former is the conventional method for RNA extraction, like TRIzol (Invitrogen), which separates RNA from DNA after extraction with acidic solution consisting guanidinium thiocyanate, sodium acetate, phenol, and chloroform. The latter solid-phase nucleic acid extraction can be found in most of the commercial extraction kits available in market, which is normally performed by using a spin column or magnetic bead. The Virus DNA/RNA Kit (GBCBIO) used in this study, and most extensively used kits, such as Qiamp DSP Virus Spin kit (Qiagen) and MagMax viral RNA isolation Kit (ThermoFisher), are all based on solid-phase nucleic acid extraction. For these solid-phase nucleic acid extraction kits, the high salt concentration is usually required for RNA binding to the solid-phase during the extraction process (Tan and Yiap, 2009). Adding AS to the viral transport medium could increase the salt concentration and acidity, which is theoretically helpful for RNA extraction. Therefore, AS has the potential to be compatible with other similar RNA extraction kits, although more research is needed to confirm it.

### 3.2 Inactivation effects on enveloped and non-enveloped viruses

To compare the virus inactivation effects of denaturing transport media containing low concentrations of GITC and 1 M AS with a commonly used denaturing transport medium, a plaque reduction assay designed for enveloped and non-enveloped viruses was performed. SARS-CoV-2 is an enveloped virus. However, the biosafety hazards relating to conducting experiments with a live SARS-CoV-2 virus caused us to use a common human coronavirus, HCoV-OC43, to evaluate the inactivation effect of various transport media against this virus. HCoV-OC43 is a human-infecting coronavirus mostly known to cause mild respiratory symptoms. It belongs to the betacoronavirus genus as well as SARS-CoV-2. We also tested HSV-1, another enveloped virus, and EV71, a non-enveloped virus with a typical icosahedral capsid structure. After subjecting the viruses to various denaturing solutions at RT for 1 h, each virus was subjected to a plaque reduction assay.

**Figure 2:**
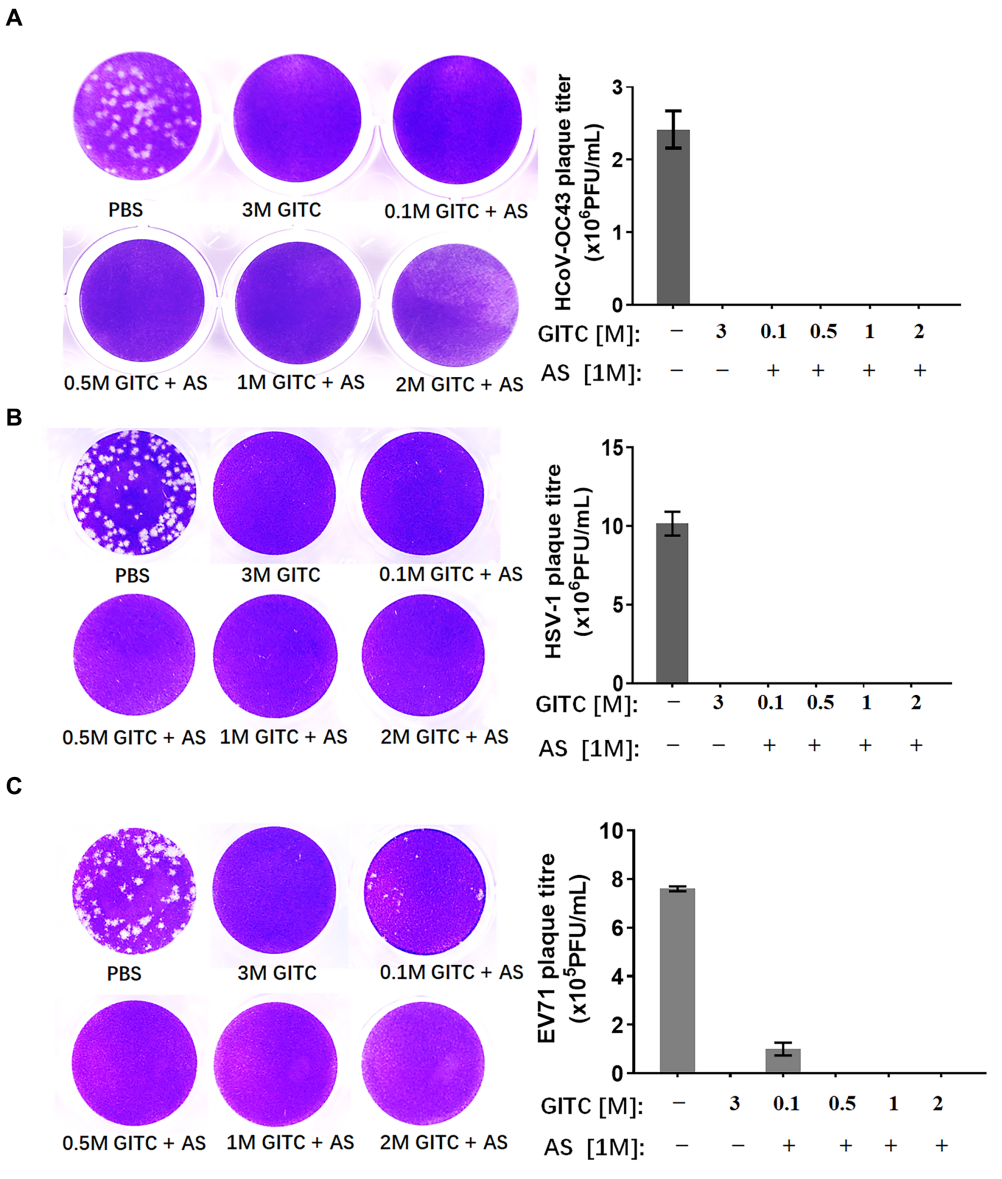
Effects of denaturing transport media containing low guanidinium isothiocyanate (GITC) and ammonium sulfate (AS) supplementation on virus inactivation. **(A)** Plaque assay targeting the human coronavirus HCoV-OC43. **(B)** Plaque assay targeting the enveloped HSV-1 virus. **(C)** Plaque assay targeting the EV71 non-enveloped virus. Viruses were incubated in PBS or transport media containing the indicated components, followed by plaque assays on RD or Vero cells. Plaques were visualized by crystal violet staining. Left panels: representative plaques. Right bar charts: virus titers calculated from the plaque assays. Data represent the average ± SD from three independent experiments.

The results showed that a common denaturing viral transport medium containing 3 M GITC completely inactivated all HCoV-OC43, HSV-1, and EV71 (Figure 2). No difference was observed in HCoV-OC43 or HSV-1 inactivation when 1 M AS was added to the media even when the GITC concentrations were reduced to 0.1 M (Figures 2A,B). In the EV71 inactivation experiments, the effect of supplementing the transport media with 0.5 M GITC plus 1 M AS was equal to that where 3 M GITC was present and EV71 was completely inactivated (Figure 2C). These results suggest that decreasing the GITC concentrations but adding AS to the transport media did not affect the virus inactivation effect when compared with general denaturing transport medium containing GITC at high concentration.

### 3.3 Effects of the test solution on RNA stability

**Figure 3:**
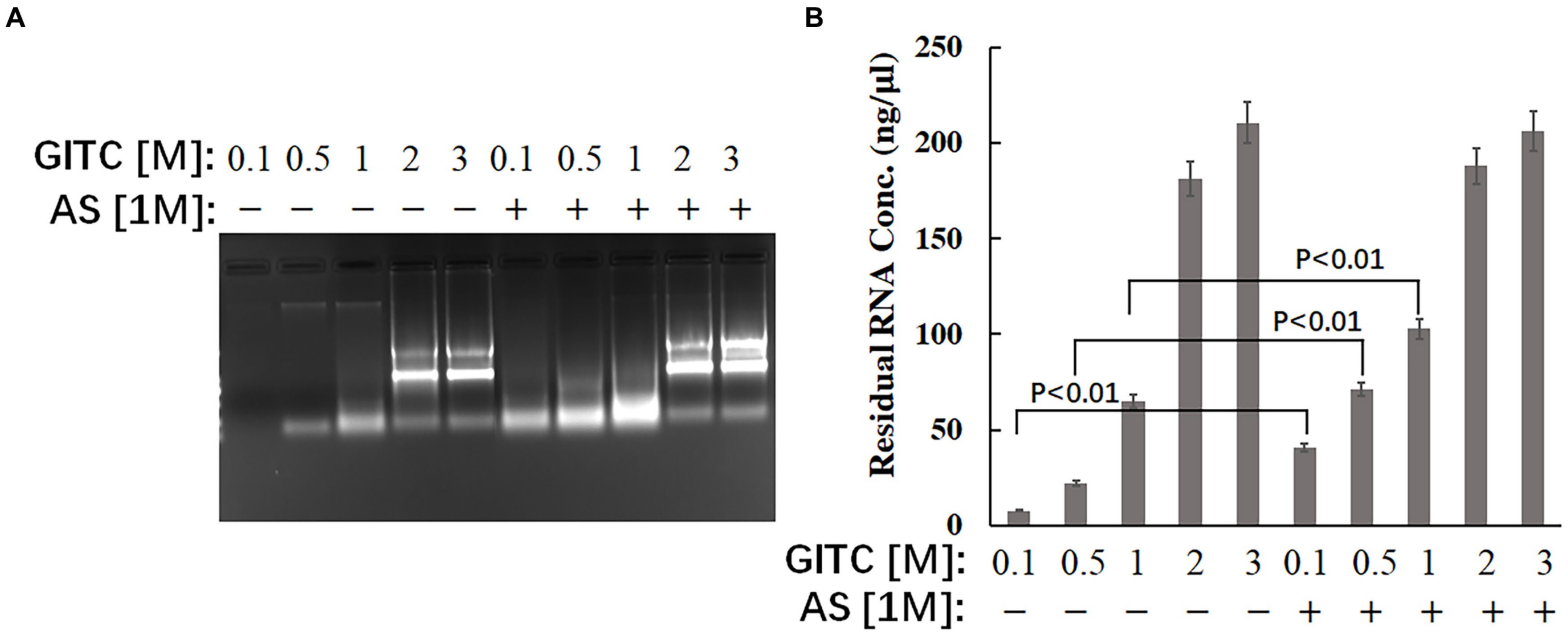
Effects of denaturing transport media containing low guanidinium isothiocyanate (GITC) and ammonium sulfate (AS) supplementation on RNA stability. The same amounts of total RNA isolated from eukaryotic cells were added to different test solutions, and the residual RNA in the mixtures was detected after incubation at RT for 24 h. **(A)** The mixtures were analyzed by 1.2% (wt/vol) agarose gel electrophoresis and visualized by staining with 4S Green Plus Nucleic Acid Stain and ultraviolet transillumination. **(B)** The concentration of residual RNA in the mixture was determined by spectrophotometer based on the absorbance at 260 nm.

The effect of the test solutions on RNA stability was evaluated to explore the possible mechanism of improved RNA detection efficiency by adding AS to the transport media. The same amounts of RNA were added to different solutions, and the residual RNA in the mixtures was detected after incubation at RT for 24 h. The results showed that adding AS to the solution could significantly enhance the stability of RNA when GITC at low concentrations (Figure 3). The residual RNA in solution with 0.1 M GITC plus 1 M AS was around six times higher than that with 0.1 M GITC but without AS (Figure 3B). This result suggests that the improved RNA detection efficiency observed in this study is related to the improved RNA stability by adding AS.

### 3.4 The compatibility of the test solution with SARS-CoV-2 antigen detection

**Figure 4:**
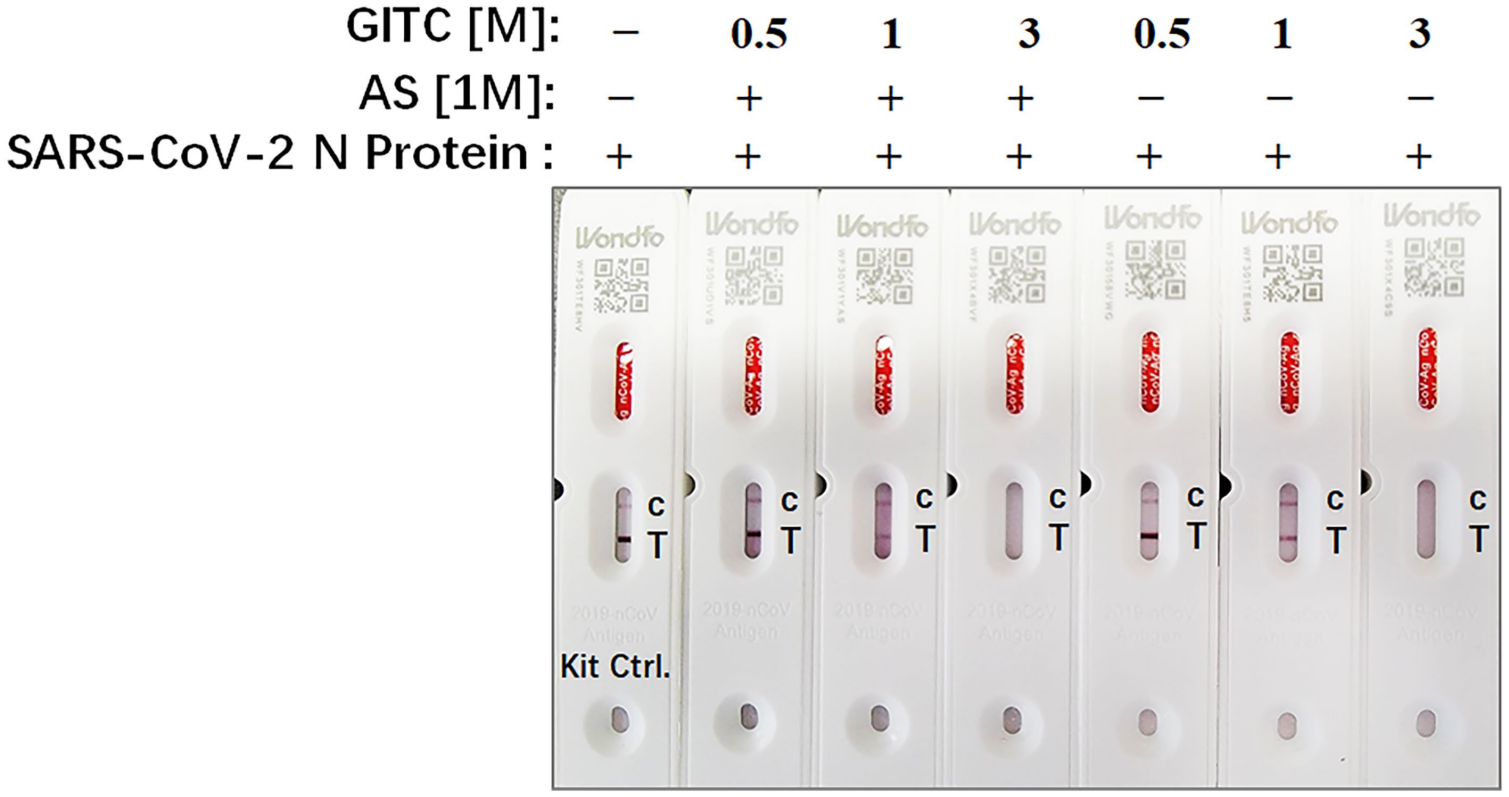
The compatibility of the denaturing transport media containing low concentrations of guanidinium isothiocyanate (GITC) and ammonium sulfate (AS) supplementation with SARS-CoV-2 antigen detection. SARS-CoV-2 N recombinant protein was diluted with PBS to 0.5 mg/mL, and then 2 μL of diluted N recombinant protein was added to 400 μL of each denaturing solution or the solution from 2019-nCoV Antigen Test Kit. Each solution was mixed and incubated for 1 min at RT. After incubation, 100 μL of the mixture was dropped into the testing device from the antigen test kit. After waiting for 15 min as recommended, read the results.

Rapid antigen tests usually lack a virucidal extraction buffer (Conzelmann et al., 2022), creating a risk of infection during antigen test performance. To examine whether the test solution in this study, which contains AS and low concentrations of GITC, is compatible with SARS-CoV-2 antigen detection, SARS-CoV-2 N recombinant protein was added to the solution and detected by a colloidal gold-based commercial 2019-nCoV antigen test kit. The result showed that the antigen in a solution containing 3 M GITC could not be detected by the kit whether adding AS or not (Figure 4). But when the concentration of GITC in the solution decreased to 1 or 0.5 M, the antigen could be detected by the kit, and there is no difference when compared to the result of the solution from the kit itself (Figure 4). This result suggests that the denaturing transport media with low GITC concentration is compatible with SARS-CoV-2 antigen detection.

## 4 Conclusion

Faced with the ongoing global COVID-19 pandemic, we sought a safer, low-cost denaturing viral transport medium for preserving SARS-CoV-2 RNA without compromising the effectiveness of viral detection. Based on the components of the modified commercial transport medium we used as a reference reagent, we reduced the GITC concentrations in the media, added AS, and obtained a new formula. We tested the different transport media by detecting SARS-CoV-2 pseudovirus in a throat swab sample and via a viral inactivation assay targeting both enveloped and non-enveloped viruses, which includes a common human coronavirus that is closely related to SARS-COV-2. We found that adding AS to the denaturing transport media reduced the use of GITC, improved SARS-COV-2 RNA detection, and did not compromise the virus inactivation effect of the media. To explore the possible mechanism, we further tested the effect of the test solutions on RNA stability and found that the improved RNA detection efficiency observed in this study is related to the improved RNA stability by adding AS. In addition, because current rapid antigen tests usually lack a virucidal extraction buffer, antigen detection was done to examine whether our denaturing transport media is compatible with SARS-CoV-2 antigen detection. We found that reducing the amount of GITC in denaturing viral transport media enables it to be compatible with SARS-CoV-2 antigen detection. The optimum concentration of GITC and AS in the denaturing viral transport medium is 0.5 and 1 M, respectively. Based on the observed results, the recommended formula for the denaturing viral transport medium is as follows: 0.5 M GITC, 1 M AS, 25 mM sodium citrate, 0.5% SLS (sodium lauryl sulfate), and 20 mM ethylenediaminetetraacetic acid.

Ammonium sulfate is a low-cost inorganic salt, which is often used for protein precipitation and purification due to its high water solubility and biosafety (Feng et al., 2024). The potential side effect of AS used for nucleic acid detection is that it may cause protease precipitation in the PCR system when the virus transport medium containing AS is directly used for nucleic acid amplification without RNA extraction. But in this case, the concentration of AS added to the nucleic acid amplification system along with the template is much lower than the concentration of protein precipitation caused by AS. Furthermore, it was reported that addition of a final concentration of 10 mM AS to the PCR buffer of four selected brands improved detection efficiency (Seifi et al., 2012). For antigen detection, there is evidence to suggest that 1 M AS does not cause significant protein precipitation (Duong-Ly and Gabelli, 2014). Therefore, no significant side effects of adding AS to the denaturing viral transport medium at the suggested concentration have been observed so far.

These findings suggest that AS is a potential component that, when added to a denaturing transport medium, may reduce the cost and improve the detection efficiency of SARS-COV-2 RNA. At the same time, reducing the amount of GITC enables the denaturing medium to be compatible with SARS-CoV-2 antigen detection, which can help reduce the risk of infection during assay performance.

## Data availability statement

The original contributions presented in the study are included in the article/supplementary material; further inquiries can be directed to the corresponding authors.

## Ethics statement

Ethical approval was not required for the studies on animals in accordance with the local legislation and institutional requirements because only commercially available established cell lines were used.

## Author contributions

GL: Formal analysis, Funding acquisition, Supervision, Writing – original draft, Writing – review & editing. JX: Investigation, Writing – original draft. YH: Investigation, Writing – original draft. WY: Investigation, Writing – original draft. JL: Investigation, Writing – original draft. RY: Investigation, Writing – original draft. QL: Investigation, Writing – original draft. XZ: Investigation, Writing – original draft. YC: Investigation, Writing – original draft. HJ: Formal analysis, Writing – review & editing. XL: Formal analysis, Writing – review & editing. KZ: Formal analysis, Writing – review & editing. ZH: Formal analysis, Writing – review & editing. QZ: Funding acquisition, Project administration, Supervision, Writing – original draft, Writing – review & editing.
